# Contribution of Neuraminidase to the Efficacy of Seasonal Split Influenza Vaccines in the Ferret Model

**DOI:** 10.1128/jvi.01959-21

**Published:** 2022-03-23

**Authors:** Miruna E. Rosu, Adinda Kok, Theo M. Bestebroer, Dennis de Meulder, Elwin P. Verveer, Mark R. Pronk, Lennard J. M. Dekker, Theo M. Luider, Mathilde Richard, Judith M. A. van den Brand, Ron A. M. Fouchier, Sander Herfst

**Affiliations:** a Department of Viroscience, Erasmus Medical Centre, Rotterdam, the Netherlands; b Department of Neurology, Erasmus Medical Centre, Rotterdam, the Netherlands; c Division of Pathology, Faculty of Veterinary Medicine, Utrecht University, Utrecht, the Netherlands; St. Jude Children's Research Hospital

**Keywords:** influenza virus, neuraminidase, NI antibodies, inactivated vaccines, ferret

## Abstract

Seasonal influenza vaccination takes into account primarily hemagglutinin (HA)-specific neutralizing antibody responses. However, the accumulation of substitutions in the antigenic regions of HA (i.e., antigenic drift) occasionally results in a mismatch between the vaccine and circulating strains. To prevent poor vaccine performance, we investigated whether an antigenically matched neuraminidase (NA) may compensate for reduced vaccine efficacy due to a mismatched HA. Ferrets were vaccinated twice with adjuvanted split inactivated influenza vaccines containing homologous HA and NA (vacH3N2), only homologous HA (vacH3N1), only homologous NA (vacH1N2), heterologous HA and NA (vacH1N1), or phosphate-buffered saline (vacPBS), followed by challenge with H3N2 virus (A/Netherlands/16190/1968). Ferrets vaccinated with homologous HA (vacH3N2 and vacH3N1) displayed minimum fever and weight loss compared to vacH1N1 and vacPBS ferrets, while ferrets vaccinated with NA-matched vacH1N2 displayed intermediate fever and weight loss. Vaccination with vacH1N2 further led to a reduction in virus shedding from the nose and undetectable virus titers in the lower respiratory tract, similarly to when the homologous vacH3N2 was used. Some protection was observed upon vacH1N1 vaccination, but this was not comparable to that observed for vacH1N2, again highlighting the important role of NA in vaccine-induced protection. These results illustrate that NA antibodies can prevent severe disease caused by influenza virus infection and that an antigenically matched NA in seasonal vaccines might prevent lower respiratory tract complications. This underlines the importance of considering NA during the yearly vaccine strain selection process, which may be particularly beneficial in seasons when the HA component of the vaccine is mismatched.

**IMPORTANCE** Despite the availability of vaccines, influenza virus infections continue to cause substantial morbidity and mortality in humans. Currently available influenza vaccines take primarily the hemagglutinin (HA) into account, but the highly variable nature of this protein as a result of antigenic drift has led to a recurrent decline in vaccine effectiveness. While the protective effect of neuraminidase (NA) antibodies has been highlighted by several studies, there are no requirements with regard to quantity or quality of NA in licensed vaccines, and NA immunity remains largely unexploited. Since antigenic changes in HA and NA are thought to occur asynchronously, NA immunity could compensate for reduced vaccine efficacy when drift in HA occurs. By matching and mismatching the HA and NA components of monovalent split inactivated vaccines, we demonstrated the potential of NA immunity to protect against disease, virus replication in the lower respiratory tract, and virus shedding in the ferret model.

## INTRODUCTION

Influenza viruses continue to be a major concern for global health, as a result of the yearly epidemics associated with substantial morbidity and mortality (1) and the imminent threat of a new influenza virus pandemic. Vaccination remains the best available preventive measure to mitigate the burden of both epidemic and pandemic influenza. The two main antigens of influenza viruses are the hemagglutinin (HA) and the neuraminidase (NA) surface glycoproteins. Although a role for NA-mediated immunity has been known for a long time (2–4), protection from influenza virus infection has predominantly been associated with antibody responses to the most abundant glycoprotein, the HA (5, 6). Consequently, current inactivated influenza vaccines (IIV) are standardized by the amount of HA in vaccine preparations and are optimized to primarily induce humoral responses to HA (7). Hemagglutination inhibition (HI) titers in serum are currently the gold standard for evaluating vaccine efficacy in individuals. Unfortunately, vaccine efficacy is occasionally hampered due to the accumulation of substitutions in the antigenic regions of HA, which leads to immune escape from preexisting antibodies (i.e., antigenic drift). As a result of this mismatch between vaccine and circulating strains, vaccines need to be updated periodically.

The influenza virus NA, the second most abundant surface glycoprotein, has been designated “the low-hanging fruit” of influenza vaccination for being one of the most accessible strategies to improve performance of current vaccines (8). HA and NA have opposing functions; while HA binds sialic acids, the receptors for influenza viruses, NA cleaves these terminal sialic acids from glycans on the host cells and virion surface. The combined activity of these proteins is important for transport of incoming virions through mucus, virus entry, and release of virions budding from infected cells (9–11). Despite 50 years of scientific evidence showing that immunity to NA plays an important role in protection against influenza infection, NA has been ignored or relegated to secondary considerations in vaccine design (2, 3, 8, 12). NA antibodies act at different stages during virus replication (12), but in contrast to HA antibodies which directly neutralize virus infection, NA antibodies are infection permissive while limiting the extent of disease. Despite these functional differences, NA antibodies have been associated with a decreased disease severity and lower virus replication and virus shedding in animal models (2, 4, 13–17). Furthermore, in human vaccination and challenge studies, NA inhibition (NI) antibody titers were shown to be an independent correlate of protection, perhaps being even more predictive than HI titers (18–20).

Like HA, NA is also subject to immune pressure from preexisting antibodies, although antigenic drift of NA is thought to occur asynchronously from that of HA (21, 22). Thus, NA immunity can be complementary and beneficial when the HA component of vaccines turns out to mismatch the circulating strains. However, the potential of IIV to induce NA-specific antibodies is poorly resolved, particularly due to inconsistencies in quantity and quality of NA in vaccine preparations which are not standardized to date (23–25).

Prompted by several recent studies demonstrating the robust role of NA in protection from seasonal influenza virus infection (18, 19, 26), we hypothesized that an antigenically matched NA protein could compensate for a reduced vaccine efficacy in the years that the HA component of vaccines does not match the HA of circulating seasonal strains. To this end, monovalent A/H3N2, A/H3N1, A/H1N1, and A/H1N2 split IIV were generated to assess the individual and combined contributions of the HA and NA proteins to protection. These vaccines were tested in ferrets for the induction of HA- and NA-specific humoral responses and their ability to reduce the viral load and prevent lower respiratory tract disease after challenge infection with an A/H3N2 influenza virus. We found that an antigenically matched NA was able to reduce disease severity, to decrease virus shedding from the nose, and to protect ferrets from lower respiratory tract infection.

## RESULTS

### Standardization of HA and NA quantities in vaccines.

**FIG 1:**
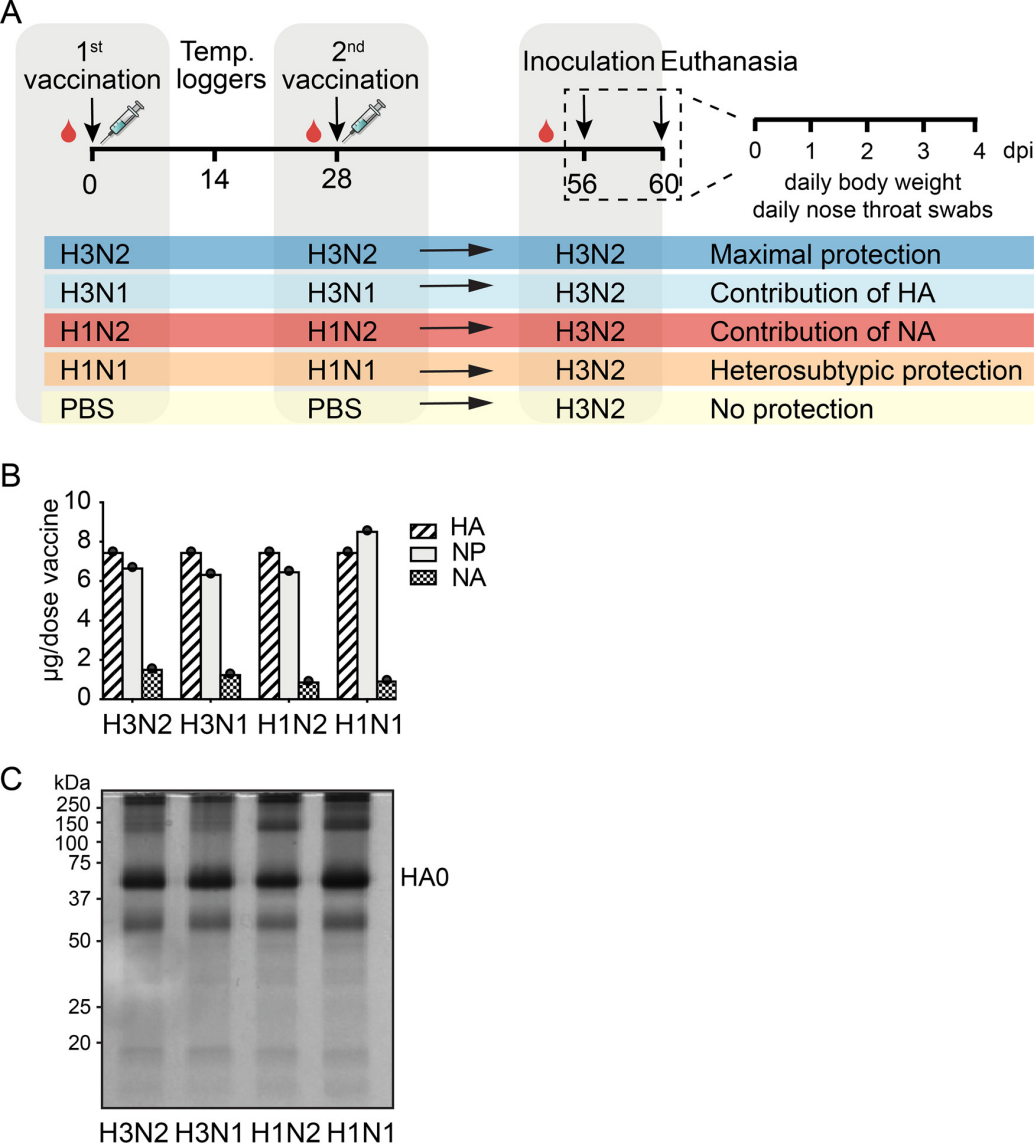
Study design and protein composition of the different split-inactivated vaccines. (A) Groups of 6 ferrets were vaccinated in a homologous prime-boost regime on day 0 and day 28 with either vacH3N2, vacH3N1, vacH1N2, vacH1N1, or vacPBS. On day 14, temperature loggers were implanted into the peritoneal cavity of the animals. Then, 4 weeks after the boost (day 56), ferrets were challenged with 10^3.9^ TCID_50_ of H3N2 virus via the intranasal and intratracheal routes and were swabbed and monitored for weight loss and body temperature daily until 4 dpi, when they were euthanized and necropsied. (B) Amount of HA, NA, and NP protein in the vaccines as determined by isotope dilution tandem mass spectrometry (IDMS). (C) Visual inspection of vaccine preparations containing 3 μg of HA protein as determined by IDMS loaded on a 10% denaturing nonreducing SDS-PAGE gel. In order, line 1, vacH3N2; line 2, vacH3N1; line 3, vacH1N2; line 4, vacH1N1. The band corresponding to HA0 was detected at around 75 kDa.

Most commercially available influenza vaccines are split IIV that primarily take the induction of HA antibody responses into account, making their efficacy very susceptible to antigenic changes in HA. To explore the potential benefits of an antigenically matched NA protein in an otherwise mismatched vaccine, several split IIV were generated containing either homologous HA and NA (vacH3N2) to assess the contribution of HA and NA to protection, only homologous HA (vacH3N1) for the contribution of HA, only homologous NA (vacH1N2) for the contribution of NA, or heterologous HA and NA (vacH1N1) to assess the possible humoral heterosubtypic immunity (Fig. 1A). The endotoxin levels in vaccines were assessed (Pierce LAL chromogenic kit; Thermo Fisher Scientific) and found to be within the acceptable levels for human vaccines (approximately 1 endotoxin unit/mL) (27). To ensure a comparable composition of all vaccines, the amount of HA, NA, and nucleoprotein (NP) was quantified by isotope dilution mass spectrometry (IDMS), for which known amounts of isotopically labeled peptides were spiked into the sample and compared to the endogenous target peptides generated by proteolytic cleavage of the target protein (28). A shotgun liquid chromatography with tandem mass spectrometry (LC-MS/MS) analysis was first used to screen vaccines for peptides suitable for quantitative analysis. Three unique peptides were selected per protein based on spectral intensity, length, and sequence conservation (Table 1). Per protein, the peptide displaying the highest recovery was ultimately selected for absolute quantification measurements. After quantification, all vaccine preparations were adjusted to contain 7.5 μg HA, which corresponded to roughly 1 μg of NA (Fig. 1B). Sodium dodecyl sulfate polyacrylamide gel electrophoresis (SDS-PAGE) analysis confirmed that equivalent amounts of protein were present in the vaccine preparations (Fig. 1C)

**TABLE 1:** Peptides selected for isotope-dilution mass spectrometry (IDMS)

Protein	Peptide^*a*^
H3	K.EFSEVEG **R** .I ^*b*^
	R.GPGSGFFS**R**.L
	K.STQAAIDQING**K**.L^*b*^
H1	R.EQLSSVSSFE**R**.F^*b*^
	R.NLLWLTG **K** .N
	R.TLDFHDSNV**K**.N^*b*^
N2	R.ILFIEEG **K** .I
	R.SGYETF**K**.V
	R.SGYSGIFSVEG**K**.S^*b*^
N1	R.TFFLTQGALLND **K** .H
	K.YGNGVWIG**R**.T
	K.YNGIITETI**K**.S^*b*^
NP	R.EGYSLVGIDPF **R** .L
	R.GVFELSDE**K**.A
	K.YLEEHPSAG**K**.D

a Underlined, peptide used for quantitation; bold, heavy isotope in labeled variants.

b Peptides described in Williams et al. (28).

### Vaccination-induced robust HI and NI antibody titers against H3, N2, and H1, but not N1.

**FIG 2:**
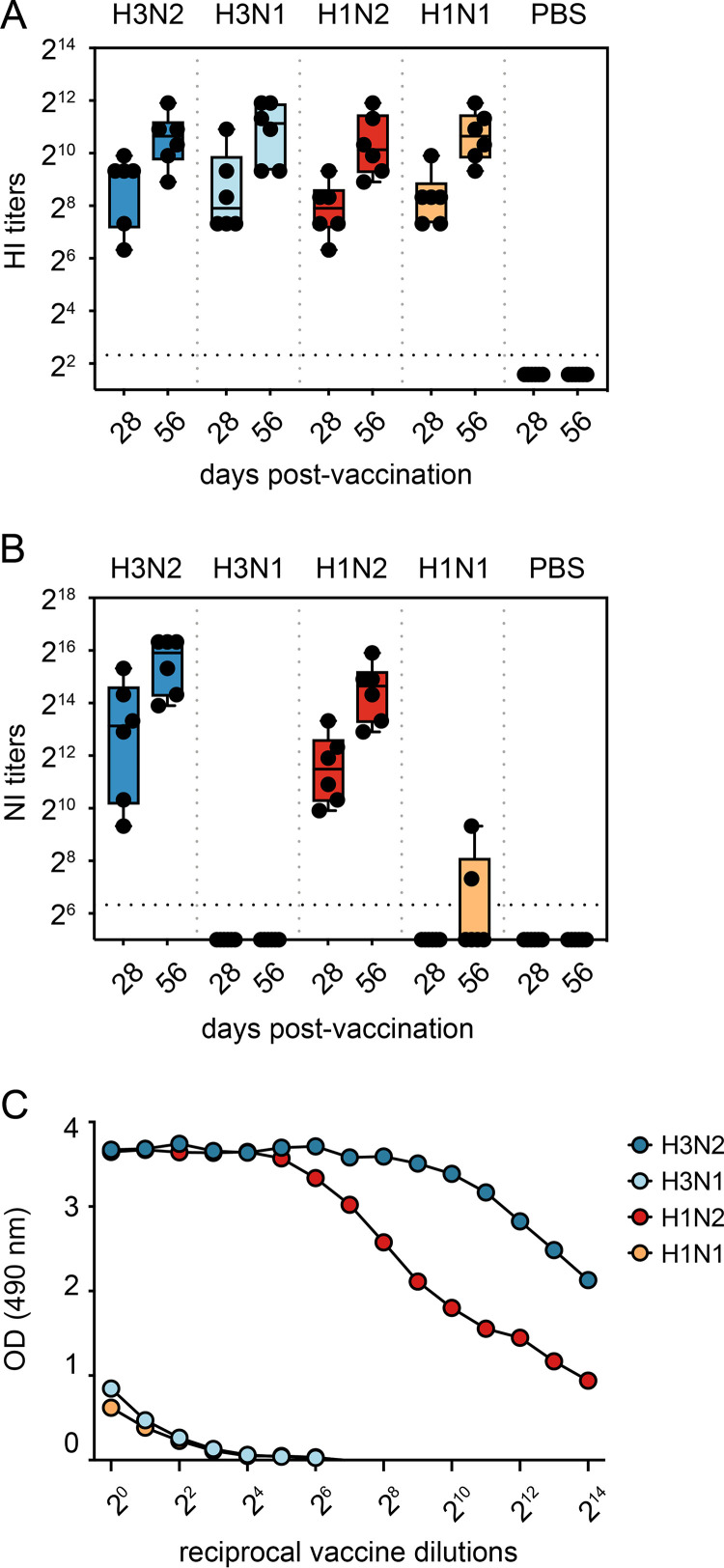
Humoral immune responses induced by vaccination. Blood was collected from the cranial vena cava of ferrets 4 weeks after each vaccination (day 28 and day 56). (A and B) Box-and-whiskers plot of antibody titers against the homologous antigen as determined by HI assay (A) and NI-ELLA (B). Black dots represent titers of individual ferrets, the box represents the interquartile range, the vertical line inside the box represents the median, and the whiskers extend to the highest and lowest observations. (C) Activity of the NA protein in vaccines measured by the ELLA assay.

**FIG 3:**
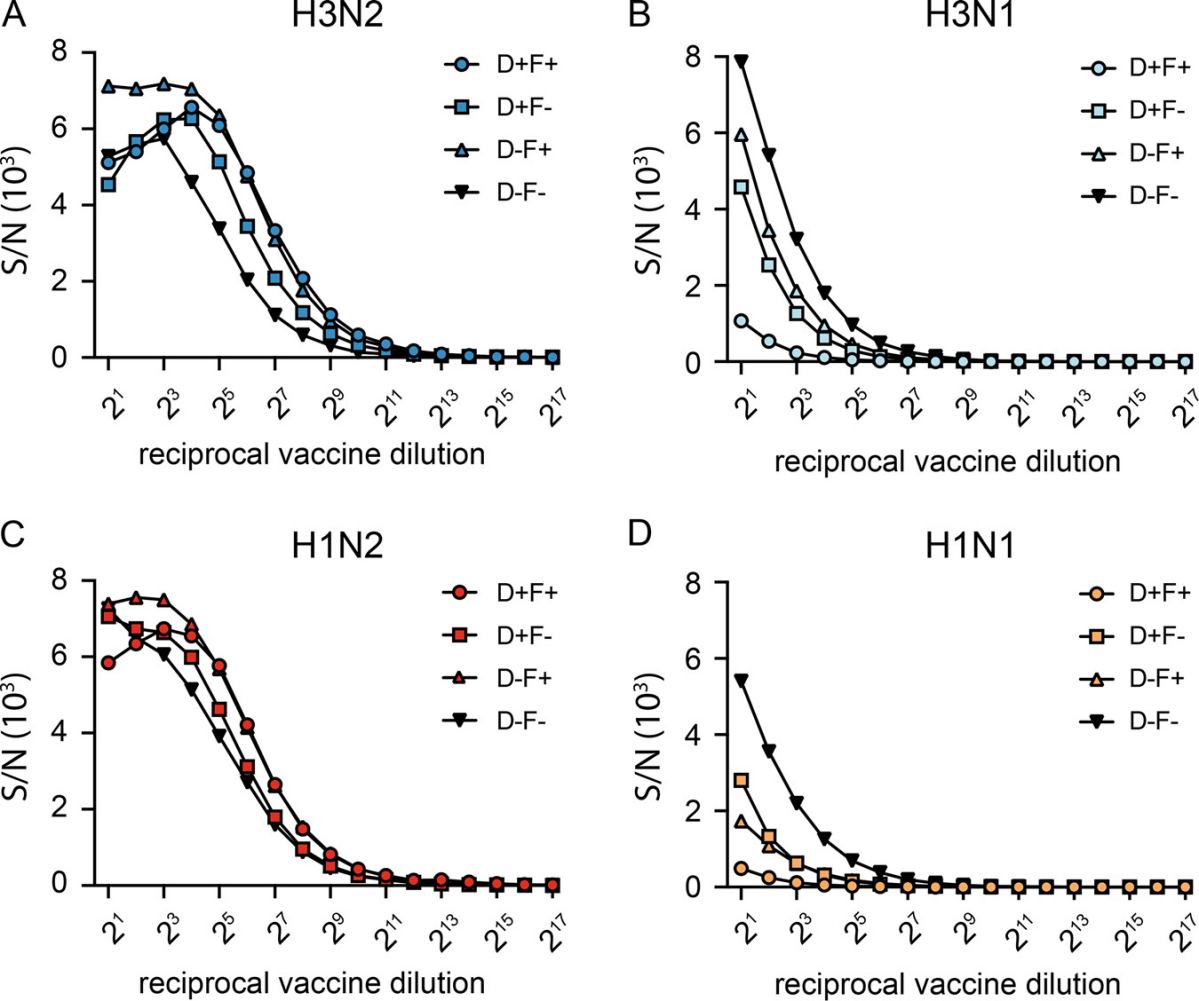
Impact of splitting and inactivation treatment on NA activity. Small batches of vaccines were produced in embryonated chicken eggs and concentrated on a 60% sucrose cushion followed by treatment with Mega10 detergent alone (D+F–), formalin alone (D–F+), both Mega10 detergent and formalin (D+F+), or none of the two (D–F–) to assess the effect of the different treatments on the activity of the NA protein in vaccines. (A to D) The activity of N2 (A, C) and N1 (B, D) in the small batches was measured using NA-Star, and the signal to noise ratio (S/N) is represented.

Ferrets were vaccinated twice, 4 weeks apart with either vacH3N2, vacH3N1, vacH1N2, vacH1N1, or phosphate-buffered saline (vacPBS) (all AddaVax-adjuvanted) and were challenged intranasally and intratracheally with in total 10^3.9^ 50% tissue culture infectious dose (TCID_50_) of A/NL/16190/68 (H3N2) 4 weeks after the second vaccination. Two doses of adjuvanted vaccines were administered to ensure induction of robust immune responses in otherwise naive ferrets. To investigate the antibody response induced by the different vaccines, we determined the HA and NA antibody titers in ferret sera using the hemagglutinin inhibition (HI) (29) and neuraminidase inhibition enzyme-linked lectin assay (NI-ELLA) (30), respectively. All vaccines induced high levels of HA antibodies, and a clear increase in titers was observed upon the booster vaccination (Fig. 2A). Robust NI antibody responses were also detected in sera from ferrets vaccinated with N2 NA (vacH3N2 and vacH1N2), with booster responses of similar magnitude as seen for HA (Fig. 2B). Unexpectedly, N1-containing vaccines largely failed to induce measurable NI antibody responses. Only two out of six animals in the vacH1N1 group had a very low NI titer after the second vaccination, whereas NI titers were not detected in the vacH3N1 group (Fig. 2B). Since all four vaccines contained comparable amounts of NA (Fig. 1B), we investigated whether there was a difference in the retention of active NA in the different vaccines. To that end, the NA enzymatic activity was measured in each vaccine by ELLA (30). Both N1-containing vaccines (vacH3N1 and vacH1N1) had a lower enzymatic activity compared to N2 vaccines (vacH3N2 and vacH1N2) (Fig. 2C). To determine whether the treatments used during the vaccine production process had affected the stability of NA in vaccines, small batches of purified vaccine viruses with comparable protein content were exposed to different combinations of detergent (2% Mega 10) and formalin (0.01%) treatment—only formalin, only detergent, both formalin and detergent, or no treatment. In the absence of treatment, the purified N2 viruses showed higher enzymatic activity than N1 viruses in the NA-Star assay, indicating intrinsic differences in activity between NA subtypes (Fig. 3). Furthermore, both formalin and detergent treatments decreased the enzymatic activity of N1 and had a cumulative effect when combined, while this was not the case for N2. These data indicate a higher susceptibility of N1 to the treatments carried out during the vaccine production that ultimately led to a loss of enzyme activity, which may serve as a proxy for protein conformation and hence a suboptimal induction of NI antibody responses.

### Disease severity was reduced upon NA vaccination.

The ferret model was used to assess the extent of protection conferred by NA in split IIV, since they reproduce many of the clinical signs and the overall course of disease seen in humans. Vaccine efficacy was assessed by comparing (i) weight loss, (ii) fever, (iii) virus shedding, and (iv) virus replication in the respiratory tract between animals of the different vaccine groups (Fig. 1A).

**FIG 4:**
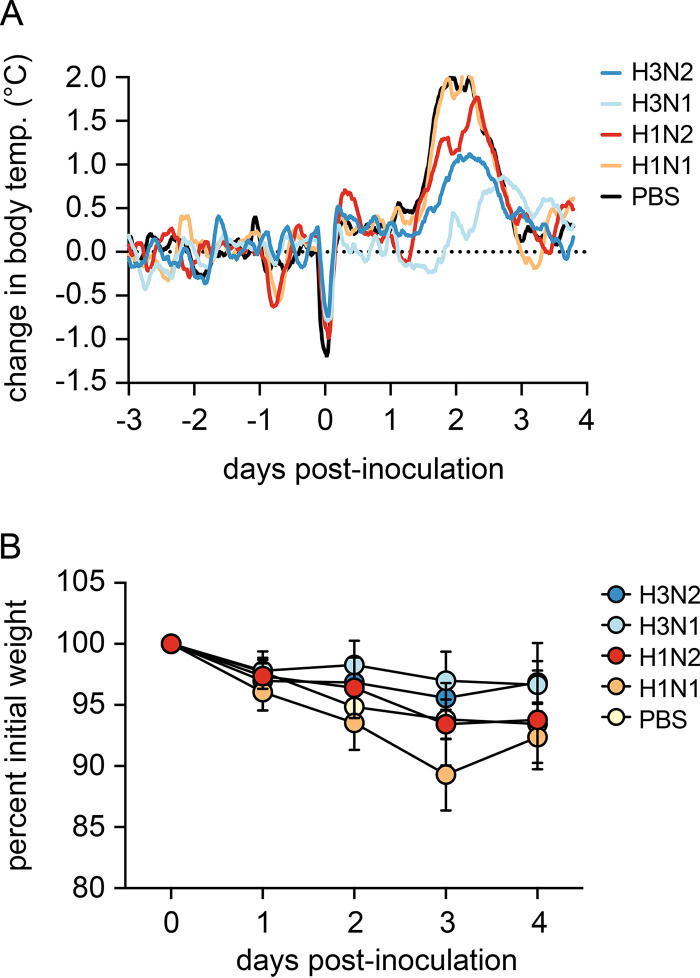
Body temperature and weight loss in vaccinated ferrets after challenge with H3N2 virus. (A) Core body temperatures were recorded every 10 min with data loggers implanted in the peritoneal cavity of the ferrets. Baseline body temperatures were calculated by averaging measurements from the 3 days prior to challenge. Sliding means over 4 h were calculated for each animal, and data were plotted as the mean per group. (B) Weight loss was registered daily and is represented as the percentage of weight compared to the day of challenge. Values are the mean and standard deviation of each group.

**FIG 5:**
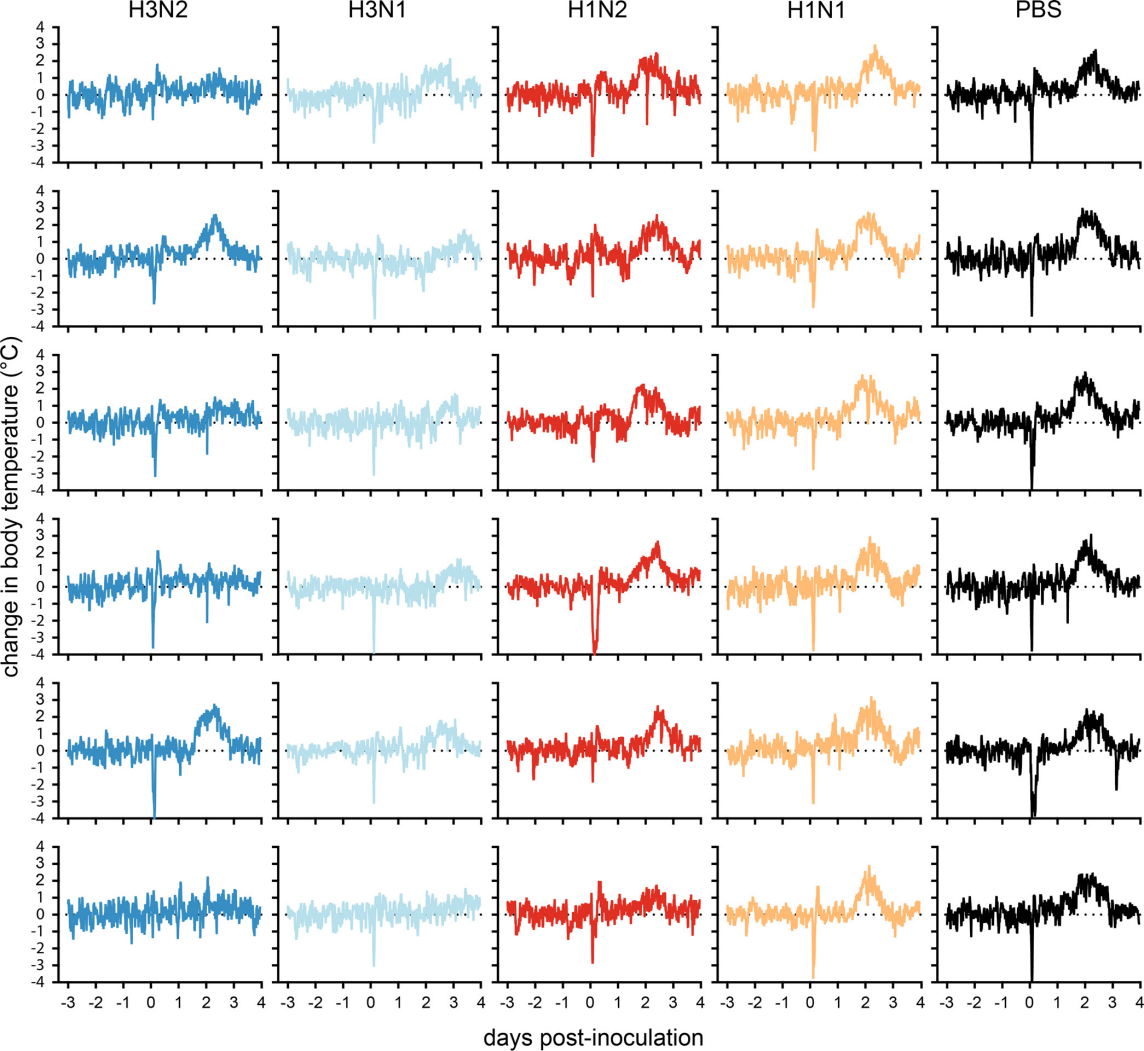
Change in body temperature from baseline in individual ferrets before and after H3N2 challenge. Core body temperatures were recorded every 10 min, and baseline body temperatures were calculated by averaging measurements from the 3 days prior to challenge. Dark blue, vacH3N2-vaccinated ferrets; light blue, vacH3N1-vaccinated ferrets; red, vacH1N2-vaccinated ferrets; yellow, vacH1N1-vaccinated ferrets; black, vacPBS-vaccinated ferrets.

Following challenge, all ferrets in the vacPBS and vacH1N1 groups displayed an increase in body temperature between 1 and 3 days postinoculation (dpi) with a fever peak at 2 dpi (Fig. 4A and 5). The extent of fever was more variable in the remaining groups, with only two and five out of six ferrets displaying fever in the vacH3N2 and vacH3N1 groups, respectively (Fig. 5). Notably, vacH3N1 animals had a slight delay in fever onset of almost a day compared to the other groups (Fig. 4A and 5). Ferrets in the vacPBS and vacH1N1 control groups displayed the highest fever, with average maximum temperatures reaching 2.2 and 2.3°C above baseline body temperature. Only ferrets in the vacH3N2 and vacH3N1 groups showed significantly reduced fever compared to vacPBS as measured by the area under the curve (AUC, *P* < 0.05 and *P* < 0.01) and reached average fever peaks of 1.6 and 1.2°C, respectively (Fig. 4A and 5). All vacH1N2 animals displayed an intermediate fever that reached on average 1.9°C above baseline.

**FIG 6:**
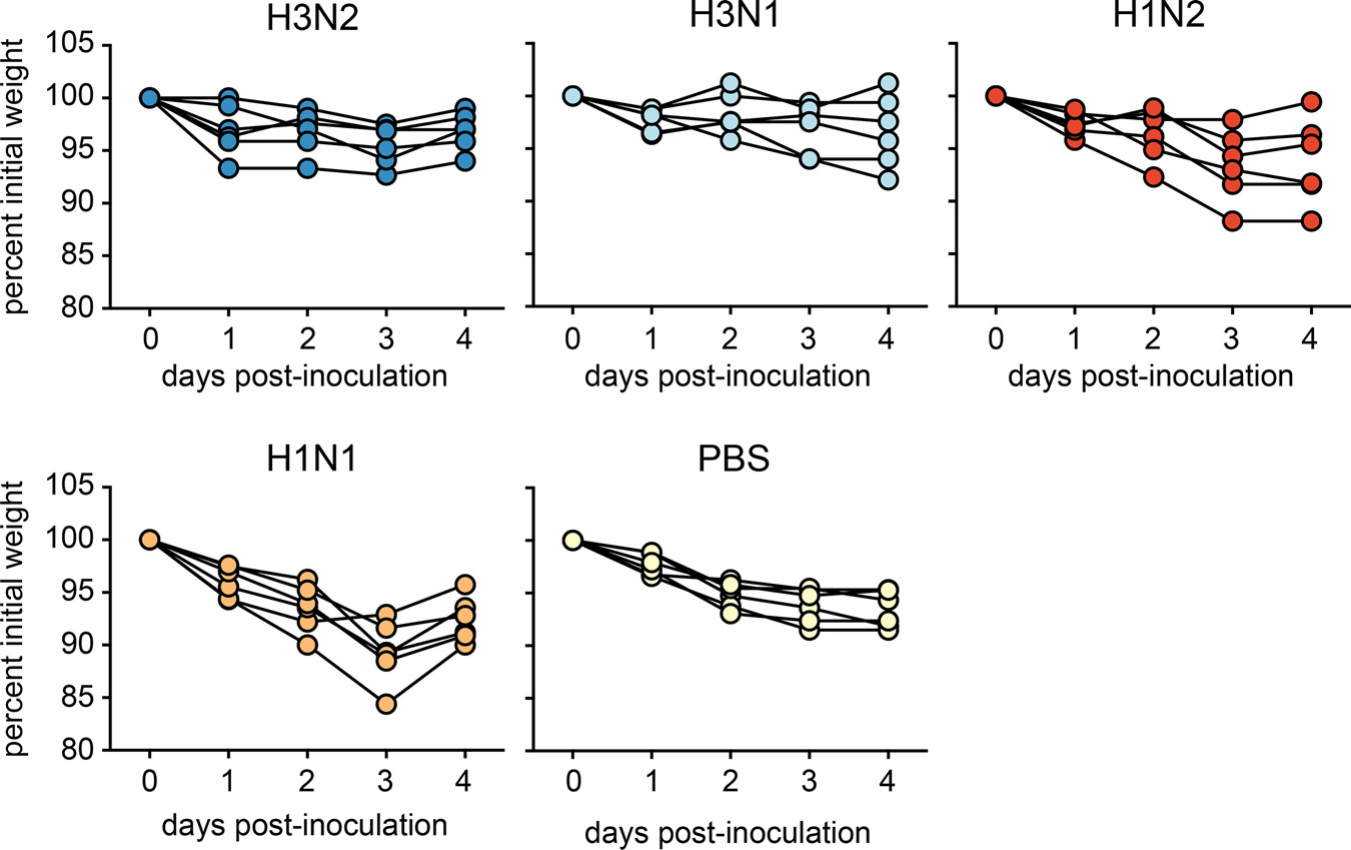
Change in body weight upon H3N2 challenge. Weight loss was registered daily and is represented for individual animals as the percentage of weight compared to the day of challenge.

There was no significant difference between the vaccinated groups and the vacPBS control group in terms of weight loss, except for vacH1N1 (AUC, *P* < 0.05). The latter experienced the greatest weight loss, with a maximum average weight loss of 11% at 3 dpi (Fig. 4B and 6). The lowest maximum average weight loss was observed for ferrets vaccinated with a homologous HA, with 4 and 3% weight loss for vacH3N2 and vacH3N1, respectively. Weight loss in vacH1N2 ferrets was less than that recorded for vacH1N1 ferrets and more variable than that of PBS-vaccinated ferrets, with a maximum average weight loss of 7% (Fig. 4B and 6).

### NA vaccination reduced virus shedding from the nose and prevented lower respiratory tract replication.

**FIG 7:**
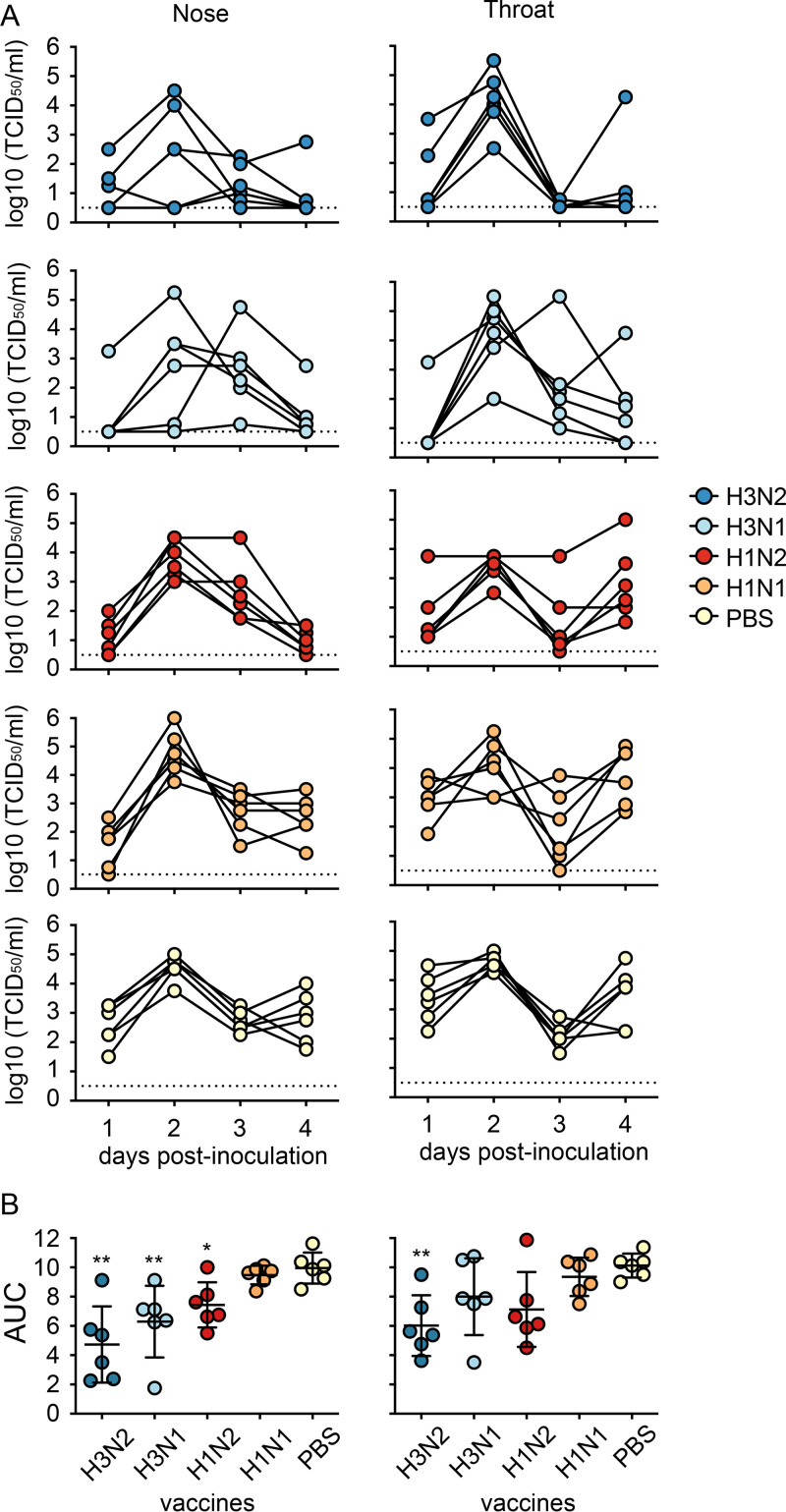
Virus shedding from the throat and nose of vaccinated ferrets upon challenge with H3N2 virus. (A) A) Virus shedding is shown as A) shedding curves for individual animals and B) the mean and standard deviation of the areas under the curve per group (AUC). Colored circles represent individual animals. Doted lines represent the limit of detection. Statistical analyses were performed using Kruskal-Wallis followed by Mann-Whitney tests with GraphPad software. **, *P* < 0.01; *, *P* < 0.05; compared to the PBS group.

Virus shedding from the throat and nose of animals in the vacPBS and vacH1N1 control groups peaked at 2 dpi with titers up to 10^5^ TCID_50_/mL and continued until 4 dpi when animals were sacrificed (Fig. 7A). Vaccination with vacH3N2, vacH3N1, and vacH1N2 significantly reduced virus shedding from the nose compared to vacPBS, as indicated by the area under the curve (AUC, *P* < 0.01, *P* < 0.01, and *P* < 0.05, respectively; Fig. 7B). This reduction was mainly due to the reduced virus shedding at 4 dpi (Fig. 7B). Although a similar trend was observed in the throat swabs, only vacH3N2 showed significantly reduced shedding compared to vacPBS (*P* < 0.01; Fig. 7B).

**FIG 8:**
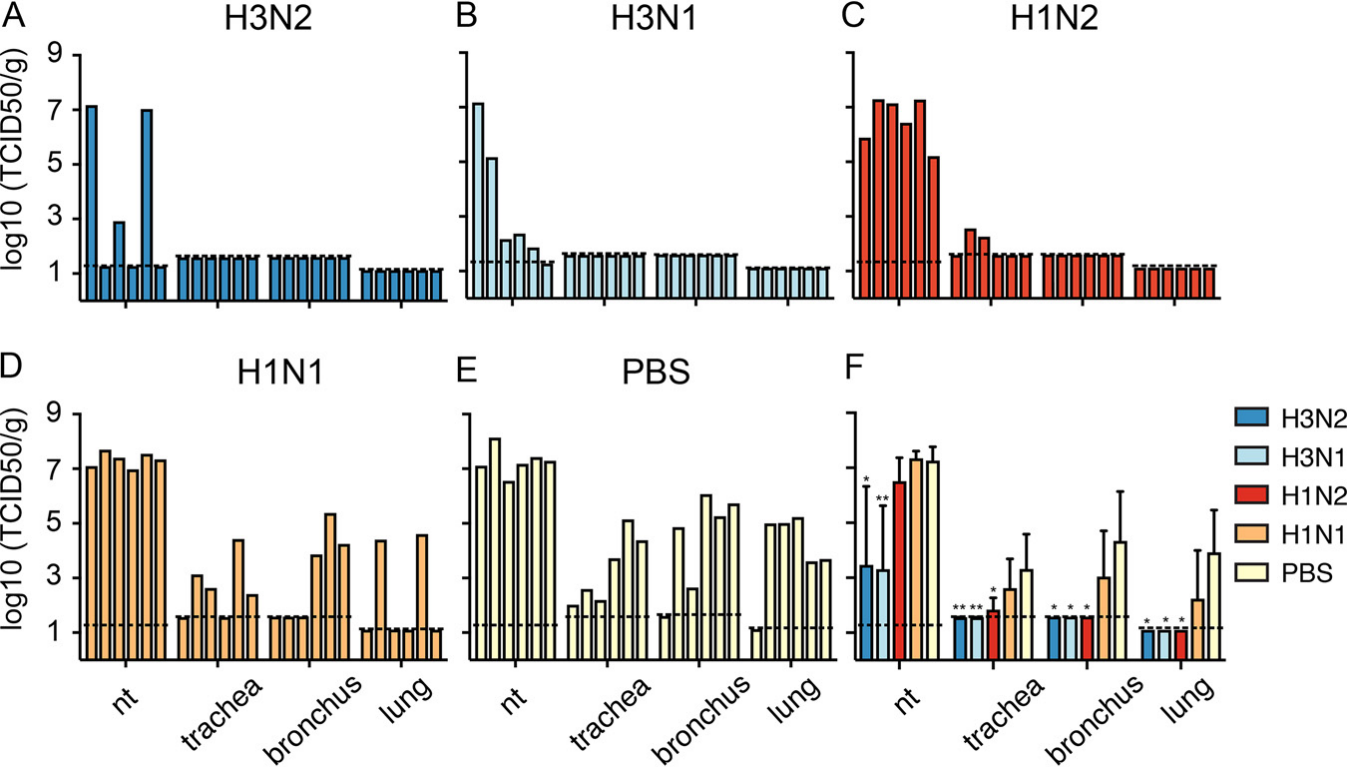
H3N2 virus replication in the upper and lower respiratory tract of vaccinated ferrets at 4 dpi. (A to F) Each bar represents titers in one individual animal (A to E) or the geometric mean with standard deviation of each group (F). Dotted lines represent the limits of detection calculated for each organ. nt, nasal turbinates. Statistical analyses were performed using Kruskal-Wallis followed by Mann-Whitney tests with GraphPad software. **, *P* < 0.01; *, *P* < 0.05, compared to the PBS group.

Four days after H3N2 challenge, animals were sacrificed and virus titers were quantified in the upper (nasal turbinates, trachea) and lower (bronchus, lung) respiratory tract to determine the protective efficacy of the different vaccines. Robust upper and lower respiratory tract infection was achieved upon H3N2 challenge as indicated by high virus titers up to 10^8^ and 10^5^ TCID_50_/g tissue in the nasal turbinates and lungs of vacPBS animals, respectively (Fig. 8E). Of note, no infectious virus was detected in the bronchus and lung of one animal of the vacPBS group, but histological analysis indicated tissue recovery from damage most likely caused by virus infection. Animals that received vacH3N2 or vacH3N1 had reduced virus titers in nasal turbinates (*P* < 0.05 and *P* < 0.01, respectively) compared to those that received vacPBS, and no infectious virus was detected in trachea, bronchus, or lung (Fig. 8A, B, and F). Similarly, no infectious virus was detected in the bronchi and lungs of animals vaccinated with vacH1N2, and only limited replication was detected in the trachea of two animals (Fig. 8C and F). So, although infectious virus was recovered from the nasal turbinates of all six vacH1N2 animals, the absence of virus in the lower respiratory tract indicated that vaccination with a matched NA can prevent the lower respiratory tract infection just as well as the homologous HA-containing vaccines (Fig. 8F). No significant differences were found between the vacPBS and vacH1N1 groups (Fig. 8F), indicating that only limited heterosubtypic protection was induced by vacH1N1 and confirming that NA alone was responsible for the protection conferred by vacH1N2.

Histopathological analysis of the lungs showed that animals in all groups displayed mild to moderate interstitial pneumonia characterized by multifocally thickened alveolar septa with infiltration of plasma cells, lymphocytes, some neutrophils, and sometimes macrophages and eosinophils, mainly associated with the bronchioles (Table 2). There was an increase in the number of alveolar macrophages in the alveolar lumina and sometimes in the conducting airways, and occasionally neutrophils were present as well. There was mild to moderate peribronchiolar, peribronchial, and perivascular cuffing and variable pneumocyte type II hyperplasia, indicating regeneration of tissue. Multifocally, there was mild exocytosis in the bronchial and bronchiolar epithelium with focal mild epithelial degeneration and necrosis and mild to severe (trachea)bronchial adenitis with degeneration and necrosis of glandular epithelial cells and variable inflammatory infiltrates as mentioned above. Apparent differences in relative lung weight, as a measure of lung inflammation, were not observed between groups (Table 2).

**TABLE 2:** Histopathology scores in ferrets inoculated with recombinant H3N2 virus^*a*^

Vaccine groups	Relative lung wt		Extent of alveolitis/ alveolar damage		Severity of alveolitis		Type II pneumocyte hyperplasia presence		Severity of bronchitis/ bronchiolitis		Severity of bronchial adenitis		Degree of peribronchial/perivascular cuffing		Severity of tracheitis		Severity of rhinitis (septum and nasal turbinates)	
	Avg	SD	Avg	SD	Avg	SD	Avg	SD	Avg	SD	Avg	SD	Avg	SD	Avg	SD	Avg	SD
vacH3N2	0.72	0.06	1.13	0.63	1.50	0.47	0.92	0.13	1.21	0.43	0.92	0.52	1.54	0.46	0.50	0.55	1.67	0.82
vacH3N1	0.73	0.07	1.63	0.31	1.83	0.58	0.79	0.33	1.13	0.21	1.17	0.41	1.46	0.40	0.50	0.55	2.33	0.52
vacH1N2	0.82	0.12	1.75	0.50	1.71	0.25	0.88	0.21	1.46	0.33	1.63	0.54	1.88	0.34	1.17	0.75	2.83	0.41
vacH1N1	0.77	0.12	1.38	0.21	1.67	0.26	0.88	0.21	1.42	0.26	1.50	0.57	1.71	0.19	1.00	0.63	2.83	0.41
vacPBS	0.76	0.09	1.67	0.38	1.75	0.27	0.96	0.10	1.63	0.31	2.13	0.34	1.88	0.21	0.33	0.52	2.80	0.45

a Histopathology scores were determined for each animal and are shown as the mean and standard deviation (SD) per group as follows. Extent of alveolitis/alveolar damage: 0, 0%; 1, 25%; 2, 25 to 50%; 3, >50%. Severity of alveolitis: 0, no inflammatory cells; 1, few inflammatory cells; 2, moderate numbers of inflammatory cells; 3, many inflammatory cells. Type II pneumocyte hyperplasia: 0, no; 1, yes. Severity of bronchitis/bronchiolitis, bronchial adenitis, tracheitis, and rhinitis: 0, no inflammatory cells; 1, few inflammatory cells; 2, moderate numbers of inflammatory cells; 3, many inflammatory cells. Extent of peribronchial/perivascular infiltrates: 0, none; 1, one to two cells thick; 2, three to ten cells thick; 3, more than ten cells thick.

In the nasal tissue of all groups, there was mild to severe infiltration of neutrophils and lymphocytes (exocytosis) in the lining of and glandular epithelium and sometimes olfactory epithelium with multifocal necrosis and with regeneration of the epithelium (hypertrophy and hyperplasia) with moderate mixed inflammatory infiltrates in the lamina propria. In the squamous epithelium in the rostral part of the nose, there were few neutrophils in the epithelium (exocytosis) and in the glands and few neutrophils and lymphoplasmacytic infiltrates in the lamina propria.

Overall, semiquantitative scoring showed that the severity of bronchitis/bronchiolitis, bronchial adenitis, and rhinitis was lowest in animals vaccinated with homologous HA (vacH3N2 and vacH3N1), although differences between groups were small. The extent and severity of alveolar damage were also lowest in vacH3N2 animals (Table 2).

Influenza virus antigen expression was often associated with the presence of histological lesions. Semiquatitative scoring showed that there was no antigen expression in the alveoli at 4 dpi in any of the groups. In the bronchioles and bronchi there was no presence of antigen-positive cells except for two animals of the vacPBS group with few bronchial epithelial cells positive for antigen and few bronchiolar epithelium cells positive, respectively (Table 3). Expression of virus antigen was mainly present in the glandular epithelium of the bronchi and the nasal respiratory, olfactory, and glandular epithelium of the nose, with the highest levels observed in vacH1N1- and vacPBS-vaccinated animals (Table 3). Few squamous epithelial cells in the rostral nose were also found positive. There were no indications of concurrent infections.

**TABLE 3:** Antigen expression in ferrets inoculated with recombinant H3N2 virus^*a*^

Vaccine groups	Bronchioles		Bronchi		Bronchial glands		Nasal glands		Septum/nasal turbinates	
	Median	Range	Median	Range	Median	Range	Median	Range	Median	Range
vacH3N2	0	NA	0	NA	0	NA	0	0–20	15	0–20
vacH3N1	0	NA	0	NA	0	NA	0	NA	0	0–10
vacH1N2	0	NA	0	NA	0	0–2.5	0	0–20	15	0–20
vacH1N1	0	NA	0	NA	0	0–7.5	10	0–10	40	0–40
vacPBS	0	0–12.5	0	0–2.5	16.25	0–32.5	10	0–10	20	0–40

a Antigen expression was determined as the median and range per group as follows. For the bronchioles, bronchi, and bronchial glands, the percentage of positively stained epithelium was estimated on every slide, and the average of the four slides was taken to provide the score per animal. For the nasal glands and septum/nasal turbinates, the percentage of positively stained epithelium was estimated on one slide per animal.

## DISCUSSION

Currently, IIVs are standardized based on the amount of HA and are primarily assessed by their ability to induce HI antibody responses. The continuous antigenic drift of HA can lead to a mismatch between the circulating strains and the vaccine strains, resulting in reduced vaccine effectiveness. In particular, the H3N2 component of the Northern Hemisphere vaccine had to be updated nine times during the 2010 to 2021 seasons due to antigenic mismatch of HA (31). The present study explores the extent to which NA immunity could compensate for a mismatch of HA and improve the effectiveness of currently available IIVs. The role of NA immunity in protection induced by influenza vaccination has been highlighted in various animal models. Mice immunized with purified NA had improved outcomes upon challenge with influenza virus and showed decreased titers and weight loss and fewer lung lesions (15, 32–34). Similar findings were also seen in other animal models, such as guinea pigs and ferrets (35, 36). NA containing virus-like particles were also found to protect against heterosubtypic H5N1 infection in mice (13). In human studies NA antibodies were shown to correlate with protection from disease (2, 4, 18–20). By assessing a series of adjuvanted split IIV containing either matched HA and NA proteins (vacH3N2), only matched HA (vacH3N1), only matched NA (vacH1N2), or mismatched HA and NA (vacH1N1) in a ferret H3N2 challenge model, we found that, in the presence of a mismatched HA, vaccination with a vaccine containing a matched NA yielded a similar level of protection in the lower respiratory tract as HA-matched vaccines.

All vaccines induced robust and comparable antibody responses to the corresponding HA proteins, with a booster effect observed after the second vaccination. NA-inhibiting (NI) antibody responses were also consistently induced by vaccines containing N2 (vacH3N2, vacH1N2), but not by those containing N1 (vacH3N1, vacH1N1). Others have shown that, while IIV are able to induce NA antibodies, the magnitude of the response may be variable, with seroconversion rates approximating 30% (26). The exact cause for suboptimal NA antibody responses is not clear, but some of the working hypotheses relate to an insufficient amount of NA in vaccines or intrinsic differences in protein stability or immunogenicity (23–25, 37). Since the amount of NA in vaccines varied by less than 2-fold, it is unlikely that the difference in antibody response toward N1 and N2 was related to protein quantity. However, the N1 was found to be less active and less stable upon vaccination formulation than N2. NA must be in its native conformation to induce functional NI antibodies. Since enzymatic activity requires a native conformation, it is often used as an indicator of NA quality and potency (37). We found that the low immunogenicity of N1, as measured in NI-ELLA, could be attributed to the low NA activity of N1 in IIV. Several studies have also shown a more robust NI antibody response toward N2 than N1 in both human and animal models that is speculated to be due to inherent differences in terms of protein stability or immunogenicity (14, 38), but it is worth noting that NA stability may be strain specific rather than subtype specific. Further studies are needed to completely understand why some strains are more stable than others during vaccine manufacturing and how to overcome this. Interestingly, the use of engineered stable recombinant N1 tetramers was shown to greatly increase immunogenicity in mice (39).

Our additional analysis revealed that N1 activity was further hampered by the formalin and detergent treatments used during the vaccine manufacturing process, while this was not the case for N2. These findings are in line with a study by Sultana et al., which found that NA stability to different treatments such as ethylenediamine tetraacetic acid (EDTA), freeze-thawing cycles, or detergents was strain specific and that enzymatic activity correlated well with immunogenicity in mice (37). Interestingly, McMahon et al. showed that the loss of enzymatic activity upon EDTA treatment did not result in loss of NA immunogenicity if the NA protein was not denatured, concluding that to induce a protective immune response, NA needs to be correctly folded but not necessarily enzymatically active (40). These observations highlight the importance of further exploring strategies to stabilize NA proteins in vaccine formulations.

As expected, vaccination with the full homologous vacH3N2 offered the highest degree of protection against challenge with H3N2, illustrated by the limited weight loss and fever, decreased virus shedding from the nose and the throat, decreased virus replication in the upper respiratory tract (URT), and complete protection against virus replication in the lower respiratory tract. The absence of a matched NA (vacH3N1) did not hinder protection if homologous HA was kept in the vaccine formulation, highlighting the strength of HI antibodies alone in IIV-mediated protection. When both HA and NA were mismatched in the vaccine, protection dropped greatly, similar to that in PBS-vaccinated animals.

Protection conferred by vaccination with homologous NA (vacH1N2) was in between that of homologous-HA vaccines (vacH3N2 and vacH3N1) and control vaccines (vacH1N1 and vacPBS). Despite having a lower efficacy, the vaccine containing a homologous NA resulted in reduced disease severity, as shown by a reduction in fever, and significantly reduced virus shedding from the nose, which may reduce onward transmission to others (41). Most importantly, protection induced by vacH1N2 in the lower respiratory tract was comparable to that induced by the fully homologous vacH3N2, as illustrated by a lack of virus replication in the lungs and bronchi of all vaccinated ferrets. The use of adjuvant and a prime-boost vaccination regime in our experiments could have affected the magnitude and breadth of immune responses induced by the split IIV, in particular the NA protein. Although this is not the vaccination strategy commonly used in humans, our findings indicate that a matched NA has the potential to protect against lower respiratory tract complications and thus severe disease. Although NI antibodies are probably the main drivers for the observed protection, we cannot exclude the contribution of antibodies exhibiting other antiviral activities, such as FcγR engagement (42–44). Protection induced by vacH1N2 was unlikely to be due to cross-reactive responses to internal virus antigens since those were derived from A/Puerto Rico/8/1934, which were also present in the vacH1N1 control.

In light of the potential for improvement of IIV, new guidelines to ensure consistent induction of NI antibody responses would be desired. Important factors to take into account are the quality and the quantity of NA in vaccine preparations. Small adjustments in vaccine formulations may largely benefit NA stability and thus immunogenicity. Provided that the quality of NA is adequate, increasing the vaccine dose was demonstrated to induce significantly more NI antibodies than a standard vaccine (45). Therefore, in future studies, the amount of NA needed to induce a protective NI antibody titer needs to be resolved. A complicating factor is that the NA protein in the vaccine may depend on the relative amount of HA and NA on the influenza viruses that are selected for each manufacturing campaign (46). In this case, supplementation of the vaccine with recombinant protein has the potential to tackle intrinsic limitations owing to the nature of the viruses used. Furthermore, although NI antibody titers of ≥40 have been correlated with reduced illness from H1N1pdm, more studies are needed to determine the antibody titer associated with protection (19). The recent use of vectored and mRNA vaccines as adopted for COVID-19 may also provide new opportunities in the influenza vaccination field (47, 48). Such vaccines could overcome the stability limitations of the NA protein, as well as the intrinsic differences in HA and NA quantities existent in current vaccines. More generally, mRNA vaccines could also allow for improved strain selection as a result of a shorter timeline for vaccine production and bypassing of egg use but remain dependent on the cold chain, which may hamper efficient global distribution.

In conclusion, our study showed that split IIV have the ability to induce robust and protective immune responses to the NA protein, although this was highly dependent on the enzymatic activity and protein stability. Ensuring induction of NI antibodies by IIVs has the potential to protect against lower respiratory tract disease and can be particularly beneficial in seasons when HA appears mismatched in the vaccine formulations. The exact role of NA immunity in the context of seasonal antigenic drift needs further investigation by using a subtype-matched, antigenically mismatched HA vaccine or challenge strain. Equally interesting would be to examine the protective role of a drifted NA in the context of an antigenically mismatched HA in vaccines. For that, it is paramount to elucidate the molecular basis for the antigenic drift of NA. This study also highlights the importance of considering the antigenic properties of NA during the yearly vaccine strain selection process to improve performance of seasonal vaccines.

## MATERIALS AND METHODS

### Cells and viruses.

293T cells were cultured in Dulbecco’s modified Eagle’s medium (DMEM; Lonza, Breda, the Netherlands) supplemented with 10% fetal calf serum (FCS; Greiner), 1% nonessential amino acids (NEAA; Lonza), 1 mM sodium pyruvate (Gibco), 2 mM glutamine (l-glu; Lonza), 100 IU/mL penicillin (PEN; Lonza), and 100 μg/mL streptomycin (STR; Lonza). Madin-Darby Canine kidney (MDCK) cells were cultured in Eagle’s minimal essential medium (EMEM; Lonza) supplemented with 10% FCS, 1% nonessential amino acids, 1.5 mg/mL sodium bicarbonate (Lonza), 10 mM 4-(2-hydroxyethyl)-1-piperazineethanesulfonic acid (HEPES; Lonza), 2 mM glutamine, 100 IU/mL penicillin, and 100 μg/mL streptomycin.

Recombinant viruses were generated according to standard reverse genetics procedures (49). Briefly, 293T cells were transfected with reverse genetics plasmids containing the internal genes of A/Puerto Rico/8/1934 and HA and NA of A/Netherlands/16190/1968 (vacH3N2), HA of A/Netherlands/16190/1968 and NA of A/Netherlands/26/2007 (vacH3N1), HA of A/Netherlands/26/2007 and NA of A/Netherlands/16190/1968 (vacH1N2), or HA and NA of A/Netherlands/26/2007 (vacH1N1) to generate the vaccine seed viruses; alternatively, all segments from A/Netherlands/16190/1968 (recombinant, wild-type H3N2) were used as challenge virus in ferrets. The supernatants from the transfections were used to inoculate MDCK cells, and viruses were harvested after 48 to 72 h. Virus stocks, ferret throat and nasal swabs and tissue homogenates were titrated on MDCK cells in the presence of tosyl phenylalanyl chloromethyl ketone (TPCK)-treated trypsin (Sigma-Aldrich). Infectious virus titers (TCID_50_/mL) were calculated from four replicates of each throat swab, nasal swab, and tissue homogenate or 10 replicates of virus stocks using the Spearman-Karber method.

### Vaccine production.

Vaccine seed viruses were plaque purified on MDCK cells and inoculated into the allantoic fluid of 11-day-old embryonated chicken eggs. Upon incubation at 37°C for 48 h, the allantoic fluid was harvested, cleared by centrifugation at 3,000 rpm (SW 32 Ti; Beckman Counter) for 10 min, and then concentrated by 2 rounds of ultracentrifugation at 27,000 rpm (SW 32 Ti; Beckman Counter) for 2 h at 4°C on 60% (wt/vol) sucrose cushions. The concentrated virus was purified on a 20 to 60% (wt/vol) sucrose gradient at 27,000 rpm (SW 32 Ti; Beckman Counter) overnight at 4°C. The next morning, the virus band was extracted and pelleted by ultracentrifugation at 27,000 rpm (SW 32 Ti; Beckman Counter) at 4°C for 2 h. The resulting pellet was resuspended in 2% Mega10 (Sigma-Aldrich) and incubated for 1 h at 37°C to allow the disruption of the virus by the detergent. The split virus was inactivated with 0.01% formalin solution for 3 days and dialyzed against PBS. The resulting split-inactivated virus preparations were stored in small aliquots at −80°C. Complete viral inactivation was confirmed by three passages on MDCK cells. Levels of endotoxin were determined using the Pierce LAL chromogenic endotoxin quantitation kit according to the instructions from the manufacturer (Thermo Fisher Scientific).

### Quantification of HA, NA, and NP in vaccines.

For influenza virus protein quantification, the split-inactivated virus preparations were initially adjusted to contain approximately 125 μg/mL of total protein concentration as determined with a Pierce bicinchoninic acid (BCA) protein assay kit (Thermo Fisher Scientific). The vaccine digests were prepared as described by Williams et al. (28) with minor modifications. Diluted vaccines (10 μl) were mixed 1:1 with 0.2% RapiGest (Waters, Bedford, MA) and then denatured for 5 min at 100°C. After cooling to room temperature, 5 μl of sequence-grade modified trypsin solution (0.4 μg/μL; Promega) was added, and samples were incubated at 37°C for 2 h. Digests were allowed to cool, and 55 μl 0.5% trifluoroacetic acid (TFA) was added to reduce the pH to 2.0 to cleave the acid labile surfactant.

The digested vaccines were analyzed by a nano-liquid chromatography (LC) Ultimate 3000 HPLC system (Thermo Fisher Scientific) coupled to the Orbitrap Fusion Lumos mass spectrometer (Thermo Fisher Scientific) to identify peptides suitable for subsequent quantification. For peptide identification, MS/MS spectra were extracted using ProteoWizard software (v3.0.19263) and analyzed with the use of a database containing the HA, NA, and NP protein sequences contained in vaccines. A set of peptides was selected based on intensity, length, and sequence conservation across variants within the same influenza virus subtype (Table 1). Selected peptides were purchased as stable isotope (SI) labeled variants in which heavy lysine 13C(6)15N(2) and arginine 13C(6)15N(4) were used (Pepscan, Lelystad, the Netherlands) (Table 1). A mix of the SI peptides at a concentration of 250 fmol/μL was prepared and used in a subsequent targeted assay (parallel reaction monitoring [PRM]).

After centrifugation at 13,000 rpm for 10 min, vaccine digests were spiked with 20 μl of SI peptide mix and measured on an Orbitrap Fusion Eclipse Tribrid MS (Thermo Fisher Scientific) coupled with a nano-LC system (Ultimate 3000; Thermo Fisher Scientific) (50). All samples were analyzed with a retention time window set to 4 min in a scheduled method of 40 min. The PRM data were analyzed using Skyline-daily (v20.1.1.32). The peaks of both the internal standard and the endogenous peptide areas were integrated based on fragment mass, expected mass error, and retention time for each peptide, and the ratio between the internal standard and the endogenous peptide was calculated. The ratios of each peptide to the amount of spiked internal standard were used to calculate the concentrations of the endogenous peptides.

### SDS-PAGE.

Vaccines were diluted 1:1 in standard dissociation buffer and denatured at 96°C for 10 min. Samples were then loaded on 10% denaturing SDS-PAGE gel, run at 100 V for 1 to 2 h on a Bio-Rad Mini-PROTEAN gel electrophoresis system, and stained with InstantBlue protein gel stain (Westburg).

### Ferret studies.

Ferret studies were carried out in strict compliance with the Dutch legislation for the protection of animals used for scientific purposes (2014, implementing EU Directive 2010/63). Research was conducted under a project license from the Dutch competent authority (license number AVD101002015340), and the study protocols were approved by the institutional Animal Welfare Body (Erasmus MC permit number 15-340-20). Influenza virus and Aleutian disease virus seronegative 6-month-old female ferrets (Mustela putorius furo), weighing 680 to 1,070 g, were obtained from a commercial breeder (TripleF, USA). Animal welfare was monitored on a daily basis. All vaccinations, implantation of data loggers, blood sampling, intranasal and intratracheal inoculations, and euthanasia were performed under anesthesia with a mixture of ketamine/medetomidine (10 and 0.05 mg kg^−1^, respectively) antagonized by atipamezole (0.25 mg kg^−1^). Swabs were taken under light anesthesia using ketamine to minimize animal discomfort. Temperature loggers were implanted in the abdominal cavity of the ferrets (DST micro-T; Star-Oddi) 2 weeks prior to the second vaccination.

### Vaccination and challenge.

Five groups of six female ferrets were vaccinated intramuscularly twice (prime-boost) 4 weeks apart with 7.5 μg of HA and approximately 1 μg of NA in 0.5 mL of the split inactivated vacH3N2, vacH3N1, vacH1N2, or vacH1N1 adjuvanted with AddaVax (1:1) (InvivoGen). The last group received PBS adjuvanted with AddaVax (vacPBS). Blood samples were collected from the cranial vena cava immediately prior to each vaccination and prior to viral challenge. Then, 4 weeks after the second vaccination, all animals were inoculated with recombinant H3N2 A/Netherlands/16190/1968, both intranasally (10^3,5^ TCID_50_/mL in 0.5 mL) and intratracheally (10^3,7^ TCID_50_/mL in 2 mL). Upon inoculation, animals were monitored daily for clinical symptoms. Body weight was recorded daily. Nose and throat swabs were collected every day and were stored at −80°C in virus transport medium consisting of minimum essential medium (MEM) Eagle with Hanks’ balanced salt solution and 25 mM HEPES (Lonza), 10% glycerol (Sigma-Aldrich), 0.5% lactalbumin hydrolysate (Sigma-Aldrich), 100 U/mL polymyxin B sulfate (Sigma-Aldrich), 100 U/mL nystatin (Sigma-Aldrich), 50 mg/mL gentamicin (Gibco), 100 IU/mL penicillin, and 100 μg/mL streptomycin mixture (Lonza) for endpoint titration in MDCK cells. At 4 days postinoculation (dpi), animals were euthanized by exsanguination under anesthesia, and tissue samples were collected for virological and pathological analysis. Challenge experiments were performed in class III isolators in a negatively pressurized animal biosafety level 3 (ABSL3) facility.

### Serological analysis.

The hemagglutinin inhibition (HI) assay was used to determine HI titers in ferret sera. Pre- and postvaccination sera were treated overnight at 37°C with receptor-destroying enzyme (RDE) filtered from cultures of Vibrio cholerae at a 1:6 ratio and inactivated at 56°C for 1 h (51). The treated sera were used in HI assays following standard procedures (29); 2-fold serial dilutions of the sera in PBS, starting at a 1:20 dilution, were mixed with 25 μL of virus containing 4 hemagglutinating units and were incubated at 37°C for 30 min. Upon incubation, 25 μL of 1% turkey red blood cells (TRBC) were added to the mixture and incubated for 1 h at 4°C. Hemagglutination inhibition titers were then read, and the HI titer was expressed as the reciprocal value of the highest dilution of serum that completely inhibited agglutination of TRBC.

The enzyme-linked lectin (ELLA) assay (30) and neuraminidase inhibition (NI-ELLA) assay were used to determine NI titers in pre- and postvaccination sera. Recombinant H6Nx viruses were titrated in ELLA to determine the optimal virus dilution that resulted in NA activity within the linear range of the titration curve (30). Once the optimal dilution was determined, the NI-ELLA was performed as described previously (30). In short, pre- and postvaccination sera were first treated overnight at 37°C with RDE followed by inactivation at 56°C for 8 h. Then, 2-fold serial dilutions of the treated sera were incubated in duplicate in fetuin-coated plates with an equal volume of diluted virus (as determined by ELLA) for 16 to 18 h at 37°C. The plates were washed and subsequently incubated with peanut agglutinin conjugated to horseradish peroxidase (PNA-HRPO; Sigma-Aldrich) at room temperature for 2 h in the dark. After incubation, plates were washed and developed with O-phenylenediamine dihydrochloride (OPD; Sigma-Aldrich). The reaction was stopped after exactly 10 min with 1 M sulfuric acid, and plates were immediately read at an optical density (OD) of 490 nm using a Tecan plate reader. The NI titers were annotated as the highest serum dilution able to block at least 50% of the NA activity.

### NA-Star assay.

The NA activity in vaccines was also determined using the NA-Star influenza neuraminidase inhibitor resistance detection kit assay (Applied Biosystems) according to the manufacturer’s instructions. Briefly, 2-fold serial dilutions of vaccine preparations were mixed in NA-Star assay buffer and incubated in a 96-well white opaque plate at 37°C for 20 min. After incubation, NA-Star substrate was added, and the plate was incubated at 37°C for an additional 30 min. Lastly, the accelerator was added, and the chemiluminescence signal was immediately read with a Tecan plate reader (F200). Both substrate and accelerator were added by two pumps that were contained in the Tecan plate reader. Samples were measured in duplicate, and the signal to noise ratios (S/N) were subsequently calculated.

### Pathology.

Tissues were fixed in 10% neutral buffered formalin (lungs after careful inflation with formalin), embedded in paraffin, sectioned at 4 μm, and stained with hematoxylin and eosin (HE) for examination by light microscopy.

Semiquantitative assessment of influenza virus-associated inflammation in the lung (four slides with longitudinal section and cross-section of cranial and caudal lobes per animal) was performed on every slide as reported previously (52); for the extent of alveolitis and alveolar damage we used the following: 0, 0%; 1, 1 to 25%; 2, 25 to 50%; 3, >50%. For the severity of alveolitis, bronchitis/bronchiolitis, tracheitis, adenitis, and rhinitis we scored the following: 0, no inflammatory cells; 1, few inflammatory cells; 2, moderate numbers of inflammatory cells; 3, many inflammatory cells. For the presence of type II pneumocyte hyperplasia we scored the following: 0, no; 1, yes. Finally, for the extent of peribronchial/perivascular infiltrates we scored the following: 0, none; 1, 1 to 2 cells thick; 2, 3 to 10 cells thick; 3, more than 10 cells thick. Slides were examined without knowledge of the treatment allocation of the animals.

For detection of influenza A virus antigen, tissues were stained with a primary antibody against the influenza A nucleoprotein (Clone Hb65; American Type Culture Collection) as described previously (41). In each staining procedure, an isotype control was included as a negative control, and a lung section from a ferret experimentally infected with pH1N1 was used as a positive control.

Semiquantitative assessment of influenza virus antigen expression in the lungs was performed as reported earlier (53); for the alveoli, 25 arbitrarily chosen fields of lung parenchyma of the 4 lung sections were examined by light microscopy (20× objective) for the presence of influenza virus nucleoprotein, without the knowledge of the identity of the animals. The cumulative scores for each animal were presented as a percentage (number of positive fields per 100 fields). For the bronchi and bronchioles, the percentage of positively staining bronchial, bronchiolar, and bronchial glandular epithelium was estimated on every slide, and the average of the four slides was taken to provide the score per animal. For the nose and trachea, the percentage of positively stained epithelium was estimated for one slide and was scored per animal.

### Statistical analysis.

Statistical analysis was performed with GraphPad v8.0 (La Jolla, CA) employing Mann-Whitney followed by Kruskal-Wallis tests; statistical significance was set at *P* < 0.05. Area under the curve (AUC) calculations were performed with R v3.6.0 using the package MESS.
